# The Effect of Route Choice in Children’s Exposure to Ultrafine Particles Whilst Walking to School

**DOI:** 10.3390/ijerph18157808

**Published:** 2021-07-23

**Authors:** Mehrdad Rafiepourgatabi, Alistair Woodward, Jennifer A. Salmond, Kim Natasha Dirks

**Affiliations:** 1 School of Population Health, Faculty of Medical and Health Sciences, The University of Auckland, Auckland 1023, New Zealand; a.woodward@auckland.ac.nz; 2 School of Environment, Faculty of Science, The University of Auckland, Auckland 1142, New Zealand; j.salmond@auckland.ac.nz; 3 Department of Civil and Environmental Engineering, Faculty of Engineering, The University of Auckland, Auckland 1142, New Zealand; k.dirks@auckland.ac.nz

**Keywords:** UFP exposure, children, walking, route choice

## Abstract

Children walking to school are at a high risk of exposure to air pollution compared with other modes because of the time they spend in close proximity to traffic during their commute. The aim of this study is to investigate the effect of a walker’s route choice on their exposure to ultrafine particles (UFP) on the walk to school. During morning commutes over a period of three weeks, exposure to UFP was measured along three routes: two routes were alongside both sides of a busy arterial road with significantly higher levels of traffic on one side compared to the other, and the third route passed through quiet streets (the background route). The results indicate that the mean exposure for the pedestrian walking along the background route was half the exposure experienced on the other two routes. Walkers on the trafficked side were exposed to elevated concentrations (>100,000 pt/cc) 2.5 times longer than the low-trafficked side. However, the duration of the elevated exposure for the background route was close to zero. Public health officials and urban planners may use the results of this study to promote healthier walking routes to schools, especially those planned as part of organized commutes.

## 1. Introduction

In recent decades, governments have been promoting walking to school using initiatives such as the Walking School Bus (WSB), wherein an organized group of children walk to school along planned routes under the supervision of adult volunteers [1]. WSBs have several benefits, including improvements in children’s physical and mental health and the reduction of cars, which contribute to air pollution around the school entrance [2]. WSBs may also help to reduce the fear of child traffic injuries amongst parents—the factor considered to be the most significant barrier to children walking to school [3,4]. WSBs can result in further benefits, including saving time, helping children to learn about road safety, and reducing stress and traffic congestion [1].

One downside of children walking to school is their potential increase in risk of exposure to traffic-related air pollution. Pedestrians are generally at a higher risk compared to those who commute by car or bus because of the longer travel time and higher ventilation required as a result of the physical exertion associated with walking [5]. Children are at particularly high risk because they breathe at a closer height to vehicle tailpipes, where the concentration of air pollutants is higher [6,7]. A study in Barcelona compared children (at 0.55 m height) and adults (at 1.70 m height), and found that children’s exposure to air pollution was 10% higher than that of adults [6]. Furthermore, children’s underdeveloped organs make them more susceptible to the adverse effects of air pollution [8].

Ultrafine particles (UFP) are defined as particles with a diameter of less than 100 nm [9]. The small size of these particles enables them to penetrate tissue and pass through different systems of the body [10]. UFP exposure may cause lung function changes, airway inflammation, enhanced allergic responses, vascular thrombogenic effects, altered endothelial function, altered heartrate and heartrate variability, accelerated atherosclerosis, and increased markers of brain inflammation [11]. There is an association between children’s health and UFP exposure, particularly among those with respiratory issues [10].

One way to ameliorate the risk of exposure to air pollution for active commuters (cyclists and walkers), especially on the planned routes to school and work, is to take routes that result in lower exposures to air pollution [12]. Active commuters tend to choose effort-optimized routes with the shortest distance or travel time [13]. However, effort-optimized routes may include highly trafficked roads associated with higher concentrations of air pollution. Choosing a route that results in a lower air pollution exposure during the daily commute could result in significant health benefits. For example, a study by Rafiepourgatabi et al. (2021) computed the health benefits of route choice using information extracted from the literature and the health risk tool AirQ+ [12]. Their results indicated that a small reduction in cyclists’ exposure to air pollution (black carbon) using a lower exposure route during the daily active commute to work or school could contribute to a reduction of up to 35 out of 100,000 deaths per year [12]. Providing information about low exposure routes may affect people’s route choice [14]. For example, a study in Antwerp, Belgium showed that when information regarding low exposure paths was provided to parents, the majority (48 out of 62) changed their children’s route to school in response [15]. 

Over the last decade, several studies have investigated the effect of route choice on people’s exposure to air pollution while travelling to their destination. For example, Broach and Bigazzi (2017) compared a cyclist’s actual route choice (which was chosen by the cyclist on their commute), the shortest route and the lowest air pollution alternative using a Land Use Regression (LUR) model [16]. They found that the inhalation dose for cyclists using the actual routes was 15% higher than the dose for the lowest pollution routes, that took on average 6% longer to travel than the shortest routes. The study in Antwerp also showed that children and parents could, on average, decrease their exposure to nitrogen dioxide by 10 $\mu g/m^{3}$ during their walk to school [15]. Molter et al. (2015) calculated children’s exposure to nitrogen dioxide and particulate matter (${\mathrm{PM}}_{10}$) using an LUR model for 100,000 arbitrary routes from various points to schools in Greater Manchester [17]. The results of the study showed that. by choosing a lower exposure route, children’s average exposure to ${\mathrm{NO}}_{2}$ and ${\mathrm{PM}}_{10}$ could be reduced by 0.40 $\mu g/m^{3}$ and 0.03 $\mu g/m^{3}$, respectively. In a similar study, Ma, et al. (2020) calculated the shortest path and the lowest dosage path from home to school for 14,091 students in Auckland, New Zealand using an LUR model [18]. They found that only 30% of the students are able to reduce their inhaled dose by more than 1% by choosing an alternative route that is no more than 1% longer than the original route. This suggests that route choice only has a small impact on air pollution dose for children walking to school.

However, these models may not provide accurate estimates of exposure because many assumptions need to be made in the construction of the models [19]. Additionally, such models are generally not suited to detecting ‘spikes’ in elevated exposures to highly variable air pollutants due to short-term events such as the passing by of a heavy-duty vehicle (HDVs) or vehicle congestion. Such events are important from the point of view of human health, as spikes in pollution can result in acute responses, particularly among children with asthma [20,21].

Several studies have directly measured active commuters’ exposure to air pollution using portable devices [19,22,23,24,25,26,27,28,29,30,31]. These studies show that avoiding highly trafficked roads (particularly those with HDVs) [19,22,23,24,25,26,27,28,29,30,31], construction sites [26,27,29], and intersections and traffic lights [22,23,24,29], as well as passing through parks and green spaces, [22,26,28,29] can reduce exposure to air pollution. The majority of these studies have focused on the exposure of adults while cycling [22,25,26,27,28,29,30,31], with only a limited number of studies measuring children’s exposure to traffic-related air pollution whilst walking to school [23,24]. Dirks et al. (2016; 2018) measured children’s exposure while walking to school, comparing two sides of a road. Dirks et al. (2016) found that maintaining distance from intersections (where possible), walking on the side of the road opposite the traffic congestion, and optimizing traffic light phases to decrease the waiting time at traffic lights may be good ways to decrease children’s exposure during their walk to school. They also found that children travelling to school could limit their exposure to air pollution and reduce exposure to short-term increases in air pollution concentration by choosing the less congested side of the road to walk along. However, neither of these studies compared two sides of a road with low traffic. Thus far, no study has compared children’s exposure to air pollution on different sides of a high-trafficked route and a quiet route.

The aim of this study is to compare three different routes to school in terms of mean exposure and the duration (in seconds) at which people are exposed to elevated concentrations (arbitrarily set to being higher than 100,000 pt/cc) of UFP. Two routes passed along a high traffic road on which the traffic flow was higher on one side compared to the other. The third route was through quiet streets. The reasons for any differences in exposure are then explored. Assessing exposure along various routes can be helpful in promoting healthy journeys to school, particularly planned routes such as WSB, which may be used to reduce children’s exposure. Lastly, policymakers could use the results of this study to encourage students, as well as parents, to walk to school.

## 2. Materials and Methods

### 2.1. Data Collection

The study took place in an urban area of Auckland, the largest city in New Zealand with a population of around 1.5 million and a density of approximately $1400\;\mathrm{people}/{\mathrm{km}}^{2}$ [32]. Air pollution from road traffic continues to be a problem in Auckland, in part because of the high rate of car ownership [33]. A high number of old vehicles in New Zealand leads to relatively high emission rates per vehicle, when compared to other developed countries [34]. Therefore, despite the favorable weather in Auckland, air pollution remains a significant issue.

**Figure 1:**
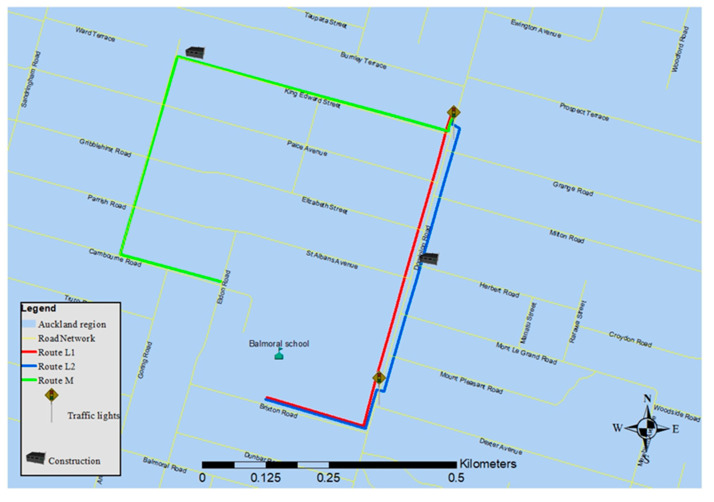
The map of the routes. Major segment of two Routes (L1 and L2) includes a high trafficked road (Dominion road), and Route M passes through the quiet streets.

Balmoral School was chosen as the school destination for this study as it is located close to Dominion Road, one of the busiest arterial roads in Auckland. The starting point of all three routes was at 371 Dominion Road, located at 850 meters from the school. Three different plausible routes from the starting point to the school were mapped. Route L1 was the shortest path to the school (Figure 1). The traffic flow on the right side of the road (immediately adjacent to Route L1) was higher than on the left side at the time at which data were collected. According to the observation (for 8 days, for the period from 8:10 a.m. to 8:40 a.m.) before data collection, the traffic flow on the left and right side of the road were 5.73 ± 1.01 and 16.96 ± 1.97 veh/min, respectively (Table 1). Route L2 passed through two crossings in parallel with Route L1, while Route M consisted of predominantly streets with low traffic. Traversing Routes L1, L2, and M took approximately 14, 15, and 20 min, respectively, walking at a pace consistent with that of a primary school-aged child (about 1 m/s).

Data collection took place between the 3 July 2020 and the 3 August 2020. Sampling began at around 8:10 am and finished at around 8:30 am, around the time when children typically walk to school and air pollution levels are expected to be at their highest. Before starting data collection each day, the clocks of all of the devices were matched, and the start and end time of each journey were noted by each of the three operators. For each day of data collection, the background concentration was measured in a quiet street, 300 m away from traffic [35,36], for five minutes immediately before starting the data collection and for five minutes immediately after finishing data collection. As suggested by Rafiepour et al. (2021) [21], the mean of the measurements was considered to be the background UFP (${\mathrm{UFP}}_{\mathrm{BG}}$) concentration. 

### 2.2. Instrumentation 

UFP concentrations, videos of the journeys, and GPS locations were captured along each route. Three P-traks (TSI Model 8525) were used to record UFP in the diameter range of 0.02–1 mm, in the concentration range from 0 to $10^{5}$ pt/cc and at 1-s resolution [37]. During sampling, the P-traks were held in the hand of the researcher (at about the height of a child’s breathing zone). Each researcher was equipped with a pocket GPS (Qstarz Q1000X [38]) device to track their location at one second intervals, with locations subsequently linked to the UFP concentration. A Samsung Galaxy S8+ [39] and a Samsung Galaxy note 10 lite [40] were used to capture videos of the roads and recordings of researchers, speaking about why any observed peaks in UFP concentration may have been occurring. 

### 2.3. Meteorology 

Meteorological parameters were retrieved from the National Institute of Water and Atmospheric Research website [41]. The closest station to the data collection site was the Auckland, Motat Ews site, located about 4 kilometers from Balmoral School. The mean ten-minute windspeed, wind direction, temperature and humidity were extracted from the database for the period of data collection (8:10 a.m. to 8:40 a.m. for each day of observation).

**Figure 2:**
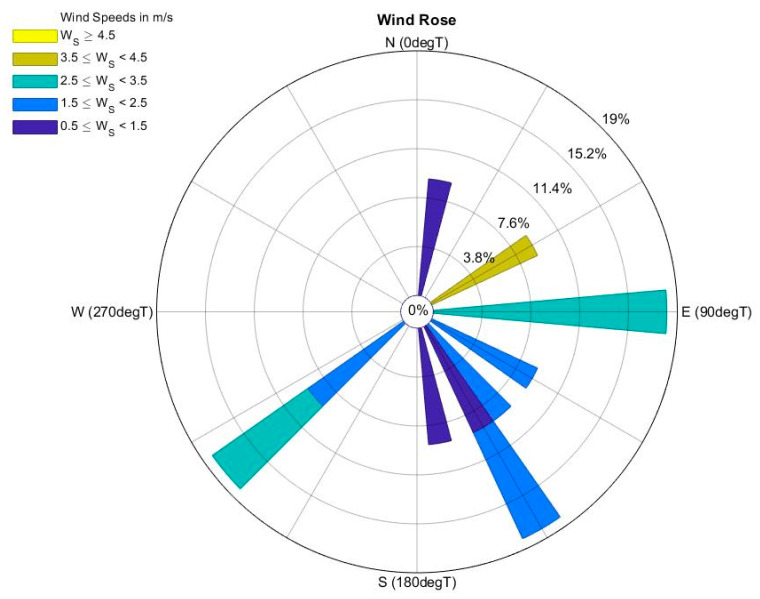
Rose plot indicating the direction (DegT) and speed (m/s) of wind using the data from the meteorological monitoring station run by NIWA (Motat EWs station).

The mean ten-minute windspeed, wind direction, temperature and humidity were extracted from the database for the period of data collection. The statistics of the meteorological data for each day of data collection are presented in Table 2. The wind speed was variable, ranging from Light air (Day 1, WS = 0.6 ± 0.36 m/s) to Gentle breeze (Day 10, WS = 4.33 ± 0.63 m/s) [42]. A rose plot created for the periods of observation (Figure 2) indicates that the three predominant wind directions during the periods of data collection were from the east, south-west and south-east.

### 2.4. Data Analysis 

The mean concentration for each route and each day was calculated. A log10 transformation was used to normalize the concentration data. A two-way ANOVA with a level of significance of 0.05 was used to investigate differences between routes regarding the mean UFP exposure. The duration of the elevated concentrations for each day of data collection was analyzed and concentrations higher than 100,000 pt/cc were classified as ‘elevated’. This is also suggested by Dirks et al. (2018). 

A generalized linear mixed model (GLMM) allows response variables from various distributions, such as a Poisson distribution, for count data, and a normal distribution for a continuous outcome (in this case, this is called a linear mixed model (LMM)) and includes both fixed and random effects [43]. This was applied (treating days of data collection as a random effect and route choice (whether L1 or L2 or M) as the fixed effect) to analyze the impact of route choice on mean exposure and the duration of elevated exposure. A growing number of studies have used GLMM in various fields including air pollution modeling. For example, Brand et al. (2019) used the same model to evaluate the association between route characteristics and time of measurement [22]. The GLMM used in this study can be seen in the following equation:

(1) $$y_{33\times 1}=X_{33\times 3}\beta_{3\times 1}+Z_{33\times 11}u_{11\times 1}+\varepsilon_{33\times 1}$$

where y is the outcome variable (33 rows = 11 day × 3 routes), X is a matrix of predictor variables (33 rows for data and 3 routes), β is the vector of fix-effect regression coefficients (three rows for three different routes), Z is the design matrix of q random effects (33 rows and 11 columns for days), and u is the vector of random effects (the 11 rows represent each of the 11 days). ε is the random effect which is not explained by the model. The main assumptions in this model are the normality of the residuals and the homogeneity of variance. 

The recorded videos were viewed to investigate the likely source of any elevated concentration. Maps were generated using Arc GIS 10.7 [44]. MATLAB [45] was used to create the graphs and perform the statistical analysis. RStudio was used to build the GLMMs and to determine the extent of validity of the models [46].

## 3. Results

### 3.1. Background UFP

**Figure 3:**
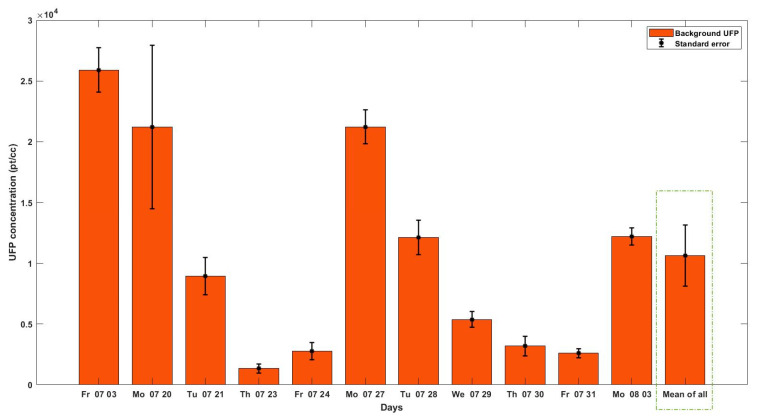
Bar plot of background UFP for each day of data collection (the error bars are the standard error of the means).

Figure 3 illustrates the background UFP for each day of data collection. The lowest background concentration (1300 ± 500 pt/cc) was recorded on Day 4. The highest amount of background UFP (25,900 ± 2600 pt/cc) was measured on Day 1. The mean background UFP was 10,600 ± 8700 pt/cc. There was a high correlation between wind speed and the background UFP ($\mathrm{Rho}=-0.74,\;p-{\mathrm{value}}_{\mathrm{ws},{\mathrm{UFP}}_{\mathrm{BG}}}\sim \;0.008$), indicating that a higher wind speed is associated with a lower background UFP concentrations. 

### 3.2. Variations in Exposure to UFP between Routes

**Figure 4:**
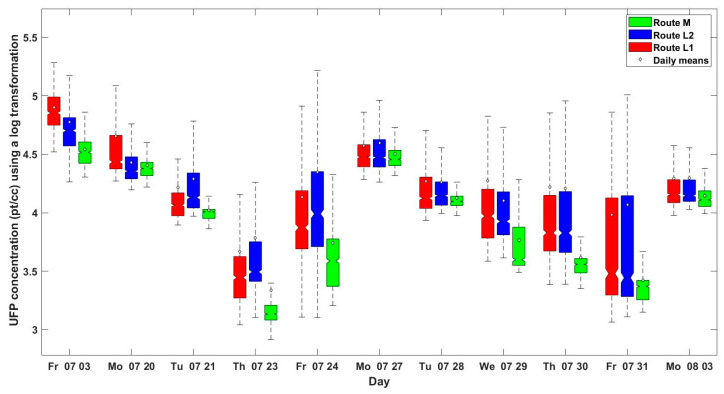
Boxplot of the collected data by day and route. Horizontal lines represent the median, boxes represent the 25th–75th percentiles and whiskers represent the 5th–95th percentiles.

A box plot of the UFP exposure (log transformed) for each route and day is presented in Figure 4. The figure illustrates that there is high variability in exposure to UFP from day to day and between routes. The maximum daily average exposures for Route L1 (79,500 ± 36,700 pt/cc), L2 (59,400 ± 38,300 pt/cc) and M (35,000 ± 11,100 pt/cc) were observed on Day 1 when the minimum wind speed (0.6 ± 0.36 m/s) and highest amount of background concentration of UFP was observed. The minimum daily average for Routes M (2200 ± 5400 pt/cc), L2 (6100 ± 9600 pt/cc) and L1 (4700 ± 7100 pt/cc) were recorded on day 4 when the wind speed was relatively high (2.7 ± 1.16 m/s) and the background UFP was the lowest. This suggests that wind speed has significant impact on the mean exposure in the routes. 

Wind speed also affected the difference between and ratio of the low exposure route to the other two routes. The correlation between wind speed and the difference between Routes L1 and M and Routes L2 and M were ($\mathrm{Rho}=0.62,\;p-{\mathrm{value}}_{\mathrm{ws},\;L1-M}\sim \;0.040$) and ($\mathrm{Rho}=0.60,\;p-{\mathrm{value}}_{\mathrm{ws},L2-M}\sim 0.048$), respectively. A high positive correlation was also observed between wind speed and the ratio of Routes L1 to M and Routes L2 to M ($\mathrm{Rho}=0.70,p-{\mathrm{value}}_{\mathrm{ws},\;L1/M}\sim \;0.017$) and ($\mathrm{Rho}=0.65,\;p-{\mathrm{value}}_{\mathrm{ws},L2/M}\sim 0.031$), respectively. This suggests that higher wind speed can lead to lower exposure in Route M compared with the two other alternatives. 

**Figure 5:**
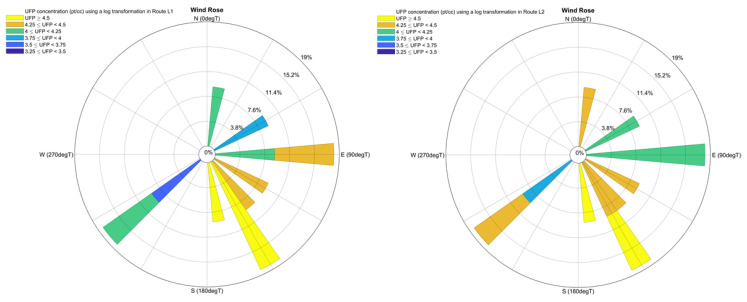
Pollution rose for routes L1 and L2 (a log transformation was used for the UFP concentration).

The pollution roses of Routes L1 and L2 can be seen in Figure 5. Winds from the north and from the southwest result in higher concentrations in Route L2 compared with route L1. In contrast, airflow from the south-east and from the east may result in higher concentrations of UFP on the Route L1. Winds from other directions have the same effect on Routes L1 and L2. This suggests that the level of UFP concentrations were similar in Routes L1 and L2 at most of the times. 

**Figure 6:**
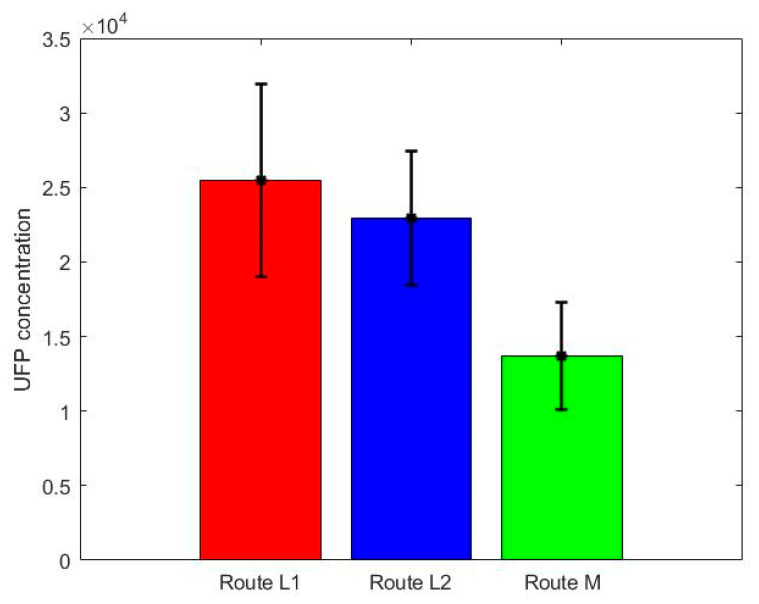
Bar plot of daily exposure for each route (the error bars are the standard error of the means).

A bar plot of the mean of daily average of each route can be seen in Figure 6. The highest mean of all daily measurements was observed on Route L1 (25,500 ± 21,400 pt/cc), explained by the higher traffic flow on Route L1 compared to the other routes. The lowest mean UFP exposure was recorded on Route M (13,700 ± 11,900 pt/cc) which included quiet streets. The average daily exposure for Route L2 was 23,000 ± 14,900 pt/cc. For each day of data collection, the mean daily exposure along Route M was lower than for the two alternative routes. 

A GLMM based on a normal distribution (as the exposure data were normalized using a log10 transformation) and identity function was used to analyze the exposure for each route. Table 3 shows the results of the model. The normality of residuals and homogeneity of variance were checked and the assumptions of the model deemed to have been met. The *p*-values show that the estimates of log transformed exposures are significant. Based on the results, an estimation of the daily mean exposures for routes L1 ($10^{4.290}$ = 19,500 pt/cc), L2 ($10^{4.288}$ = 19,400 pt/cc) and M ($10^{3.967}$ = 9300 pt/cc) was obtained. In other words, a person walking on Route L1 or L2 experienced twice the mean UFP exposure compared with the person on Route M. 

To investigate whether there was a significant difference between routes regarding exposure to UFP, an ANOVA based on the GLMM was used. The ANOVA revealed that the differences in exposure between three routes were statistically significant ($F-\mathrm{value}=606,\;p-{\mathrm{value}}_{\mathrm{df}=2}=1.2\times 10^{-15}$). A paired wise *t*-test showed that there was a significant difference between Routes L1 and M ($p-{\mathrm{value}}_{M,L1}\sim \;3.04\times 10^{-9}$), as well as Routes L2 and M ($p-{\mathrm{value}}_{M,L2}\sim \;3.96\times 10^{-9}$). However, the difference in mean exposure between Routes L1 and L2 was not significant ($p-{\mathrm{value}}_{L1,L2}\sim \;0.96$). 

### 3.3. Elevated Exposure 

**Figure 7:**
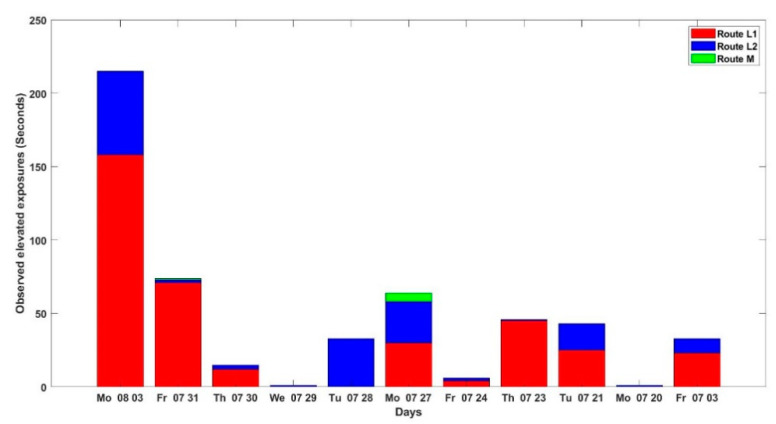
Number of elevated concentration (in seconds) per day.

A bar plot of the duration of elevated UFP exposure on each day for each route can be seen in Figure 7. In total, 531 s of elevated exposure were observed. On Routes L1, L2, and M, 368, 156 and 7 s of elevated exposure were recorded, respectively. For the majority of the days, the duration of elevated exposures in Route L1 was longer than on the other two routes. The lowest number of elevated exposures was observed on days 4 and 10. The longest duration of elevated exposure to UFP on Route L1 was observed on Day 1 when the wind speed was at its lowest (0.6 ± 0.36 m/s). 

A GLMM using a Poisson distribution and natural log function was used to analyze the differences in the duration of elevated exposures between routes. Table 4 shows the result of the GLMM. The model’s assumptions, including the normality of the residuals and homogeneity of the variance, were found to have been met. As the *p*-values are less than 0.05, the estimates of the log transformed of the duration of daily exposure to elevated concentrations for each route were found to be significant. The results demonstrate that the median duration of exposure for Routes L1, L2 and M were approximately 15.12 ($e^{2.716}$), 6.41 ($e^{1.858}$), 0.29 ($e^{-1.246}$) seconds, respectively. On the high-traffic side of the road, a person would be exposed to 2.5 times the duration of elevated exposure compared with Route L2.

An ANOVA and paired wise *t*-tests were used based on the GLMM to validate the differences found in the duration of elevated exposure across the routes. The result showed a significant difference ($F-\mathrm{value}=67,\;p-{\mathrm{value}}_{\mathrm{df}=2}=1.89\times 10^{-13}$) in duration of elevated exposure between three routes. Paired wise *t*-tests revealed a significant difference between the three pairs of routes ($p-{\mathrm{value}}_{M,L1}\sim \;0,$ $p-{\mathrm{value}}_{M,L2}\sim \;6.66\times 10^{-16},$ $p-{\mathrm{value}}_{L1,L2}\sim \;0$).

**Figure 8:**
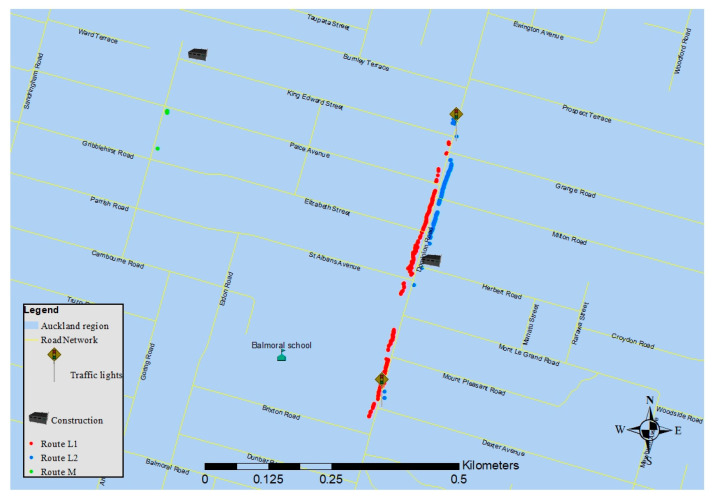
Map of the elevated exposures locations: points are well distributed for route L1. Only seven elevated exposure were observed in Route M. Most of the elevated exposures in route L2 appeared in the first segments of the route.

A map showing the locations in which elevated exposures were experienced can be seen in Figure 8. For Routes L1 and L2, all periods of elevated exposure took place along Dominion Road, the main segment of the routes. On Route M, only seven seconds of elevated exposure were observed. The majority of elevated exposures on Route M were due to a garbage truck in the street. The two traffic lights in the main segment of routes L1 and L2 caused vehicles to move in waves and played an important role in the occurrence of exposure to elevated concentrations. 

**Figure 9:**
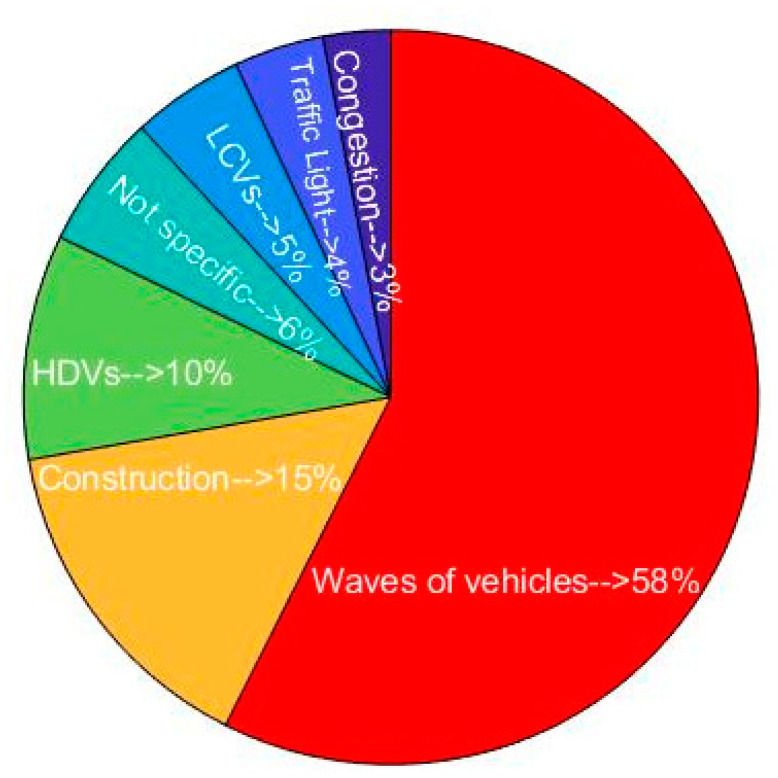
Potential reason for occurrence of elevated exposures.

By referring to the videos and operators’ notes, the reasons for the elevated exposures were found (Figure 9). The elevated UFP concentrations were categorized into seven groups including waves of vehicles (as a result of queued vehicles behind the traffic lights), construction (due to civil engineering works and construction HDVs such as backhoes and excavators), HDVs (buses, trucks and other heavy diesel vehicles), traffic lights (include crossings and waiting at the two traffic lights), and non-specific (we could not find any reason for some of the elevated concentrations). The waves of vehicles were responsible for the majority of the elevated exposures (58%), with construction activities on or near the road the second most common cause of elevated exposures (15%) and passing HDVs (such as trucks and buses) responsible for 10% of the elevated exposures. There were some instances of elevated exposure where no clear cause could be identified from the videos (6%). Light commercial vehicles (LCVs) contributed to some of the elevated exposures (5%). 

**Figure 10:**
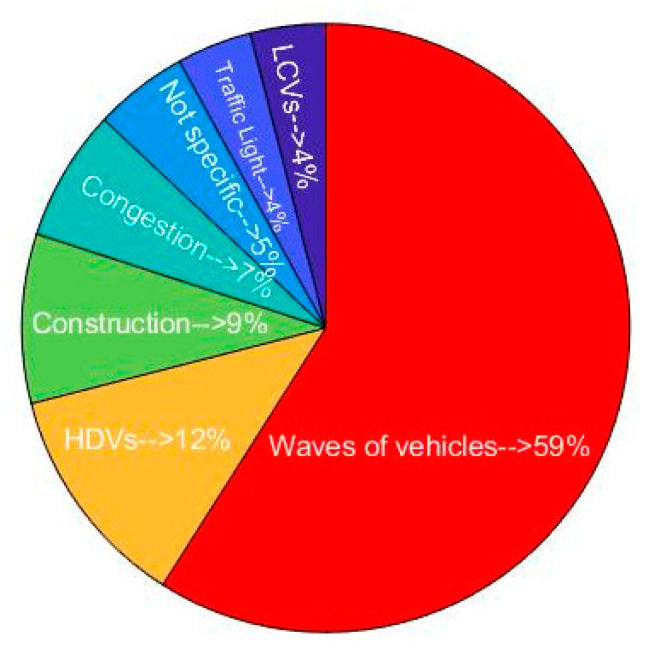
Potential reason for events resulting in elevated exposures.

In total, 56 events were associated with elevated exposures. Most events occurred on route L1 (64.03%) followed by routes L2 (30.03%) and M (5.03%). The reason for events (Figure 10) was similar to the pie chart of the duration of elevated exposure. Waves of vehicles were found to be the most important reason for elevated exposure events. Passing beside HDVs, construction, congestion, traffic lights, and LCVs caused 12%, 9%, 7%, 4% and 4% of the events, respectively. For 5% of the events, no specific reason was found. 

## 4. Discussion

In cities like Auckland, pedestrian commutes to schools may lead to exposure to traffic related air pollution [23], and even short periods of exposure to high concentrations of air pollution can cause adverse health effects, especially among children with asthma [47]. Choosing routes away from traffic may reduce mean exposure and elevated exposure. This study was designed to determine the effect of route choice on exposure to UFP while walking. The UFP concentrations were compared between three different walking routes to a school, each with different characteristics. 

The results showed a significant difference between the three routes regarding the mean exposure to UFP. Although the traffic flow on one side (16.96 veh/min and 75% of all traffic flow) was higher than the other side (5.73 veh/min which was %25 of all traffic flow), the Tukey test found no significant difference between the two routes on different sides of the high traffic road. This is in contrast with the findings by Dirks et al. (2018) who showed that the person walking on the quieter side of road (68 veh/min with a ratio of traffic of 60% on busy side to 40% on quiet side) may be exposed to a lower concentration of UFP. This could be due to the role of meteorology in UFP exposure in this study. The wind in their study was only from the west and south-west with an average speed of 3.85 ± 1.09 m/s. However, in the present study, the flow of air during the period of data collection was from various directions, including east, south-west and south-east, with an average speed of 2.14 ± 1.02 m/s. More research is required to investigate the correlation between the wind speed/direction and exposure to air pollution on both sides of the road. Other studies have also confirmed that meteorology is one of the main factors associated with exposure to UFP [23,24,29].

The quiet, residential route had a lower mean UFP exposure in comparison with the other routes (Figure 6). These findings can be attributed to the lower traffic flows on this route. As reported previously, it appears that choosing the walking route with the lowest traffic flow results in reduced exposure to air pollution [16,22,25,26,27,28,29,31]. 

A GLM model showed a significant difference between the three routes regarding the duration of exposure to elevated concentrations. The highest number of elevated concentrations was recorded on Route L1 (~15.12 s/day), followed by Route L2 (~6.41 s/day) and Route M (~0.29 s/day). This is consistent with a study by Dirks et al. (2018), in which it is mentioned that walking on the quiet side of the road can lead to a low total number of spikes in concentration. However, so far no study has compared exposure to elevated concentrations for walkers along various routes. 

Reasons for the elevated exposure to UFP concentrations were recorded. On the high traffic routes, traffic lights at intersections caused queues of idling vehicles, as well as vehicles moving in waves. Longer durations of elevated concentrations of UFP exposure were associated with high numbers of HDVs on the high traffic routes. On each of the high traffic routes there were two bus stops which also increased exposure to UFP when the buses stopped to collect and drop off passengers. Construction sites were responsible for elevated UFP exposure. These results are consistent with those of other studies, suggesting that a close distance to HDVs and exhaust plumes of passing vehicles [23,24,29,48], bus stops [49,50], and construction sites [26,29] can result in exposure to elevated concentrations and higher level of mean exposure.

The meteorological parameters, which were different from one day to another, affected the exposure to UFP. For example, when the wind speed was at its lowest, the highest daily exposure and the longest duration of elevated exposure was observed for all three routes. In this study, the difference in meteorological parameters (particularly wind speed) could be more significant than choosing sides of the road in terms of the mean exposure to UFP. Furthermore, the higher wind speed led to a higher difference and a higher ratio between the background route and the alternative routes along high traffic roads. This indicates that the wind’s effect on pedestrian exposure is higher in the routes away from traffic compared with the routes at a close distance to the source of air pollution. Therefore, regardless of meteorology, choosing the low traffic side of the road may help walkers to experience a shorter period of elevated exposures.

The results of this study provide an exciting opportunity to advance our knowledge of the effect of route choice in children’s exposure to UFP while walking to school. Previous studies compared two sides of the road, regarding children’s exposure to UFP [23,24], but did not simultaneously consider exposure on a route consisting of quiet streets. In the present study, children’s exposure to UFP on a quiet route was compared to two routes along a high traffic road. Although some studies compared routes using models (such as LUR models), and compared the cleanest route (lowest dosage) and the shortest route, the models are not accurate [19]. Additionally, they could not compare the routes regarding the duration of elevated exposure to air pollution due to short term events such as passing HDVs. However, in this study the duration of elevated exposure to UFP between the three routes was compared.

This study had several limitations. Firstly, data collection was carried out over a short period of time (one month). The meteorological measurements in this study will not have been representative of the average weather over a whole season and year. Secondly, the study was conducted in one neighbourhood and may not be representative of exposure over the whole of the city. Testing UFP exposure during other seasons and around different schools may lead to different results. Future studies could replicate this study in different locations and at different times of year.

There are several policies that can be implemented to reduce air pollution for active commuters in cities. Banning HDVs in residential areas can decrease exposure to air pollution for active commuters, as HDVs are one of the main sources of urban pollutants such as UFP and black carbon. For example, the UK government is planning to ban all diesel vehicles by 2040 to reduce air pollution [51]. Cities can also be designed to promote active commuting and decrease traffic-related air pollution exposure. For example, building separate routes away from traffic for active commuters can significantly reduce exposure to air pollution [21].

In addition, providing information regarding the least polluted route can promote healthy active commuting. Many people currently use map-based applications to choose the shortest route to their destination. However, there is potential also to provide air quality and traffic level information for each route so that individuals can choose a route based on air quality [15]. Even a small reduction in daily exposure can make a significant difference in annual exposure and thereby improve health. A study conducted in Antwerp showed that parents escorting their children to school tended to choose the cleanest route when they were provided with information about air quality, and as a result 60% of participants were exposed to less pollution [15]. We conclude that providing people with information about air quality may be an effective method for promoting healthy active travel. Transport agencies could be required to identify lower pollution routes using a spatiotemporal model of air pollution and to disseminate this information in ways that help with route mapping. Furthermore, this should involve stakeholders (including local government, local communities, experts and academics, the media, and the private sector, known as Penta Helix collaboration [52,53,54]) in better planning, implementation, and monitoring of the initiative, in order to ameliorate air pollution exposure and improve public health. 

## 5. Conclusions

Our analysis from the collected data on three WSB routes to school revealed that route choice can impact significantly on children’s exposure to UFP. Children should be encouraged to use the low traffic side of the road and avoid walking in close proximity to HDVs, long waits at traffic lights, and passing by construction sites, in order to decrease the duration of elevated exposure and reduce mean exposures.

In the absence of reliable modelling, routes with the lowest levels of air pollution can be identified using portable air quality monitoring devices to inform children and caregivers with relevant information on the impact of route choice [55]. Government may identify the factors influencing people’s exposure to air pollution (such as dense traffic and traffic lights) along their usual route, and people can be informed about these influential factors to avoid high exposure. If avoiding high traffic roads is not possible, pedestrians should be encouraged to choose the side of road with lower traffic flow to reduce their exposure to elevated concentrations of UFP. In particular, this can promote health among children with asthma, as exposure to short term events may cause adverse health effects [20]. Furthermore, governments may build infrastructure and create facilities to decrease exposure to air pollution for walkers (particularly children using WSB). Consequently, children’s exposure to air pollution can be reduced and, in the long term, population health will be improved.

## Figures and Tables

**Table 1:** Traffic flow (#vehicle/minute) on each side of Dominion road based on observation made in 2019.

Mean Traffic on the Left Side (#veh/min)	Mean Traffic on the Right Side (#veh/min)	Mean HDV’s Flow on the Right Side (#veh/min)	Mean HDV Flow on the Right Side (#veh/min)
5.73 ± 1.01	16.96 ± 1.97	0.43 ± 0.22	0.91 ± 0.43

**Table 2:** The meteorological parameters retrieved from NIWA (mean and standard deviation were obtained based on three collections of 10-min data).

	Wind Direction (Mean ± Std) (Deg)	Wind Speed (Mean ± Std) (m/s)	Temperature (Mean ± Std) (°C)	Relative Humidity (Mean ± Std) (%)
20200703 (D1)	145.33 ± 5.77	0.60 ± 0.36	1.80 ± 0.46	100.00 ± 0.00
20200720 (D2)	154.67 ± 5.03	2.03 ± 0.15	10.70 ± 0.62	94.33 ± 2.52
20200721 (D3)	13.67 ± 4.62	1.00 ± 0.10	12.50 ± 0.36	99.33 ± 1.15
20200723 (D4)	230.75 ± 7.18	2.70 ± 1.16	12.33 ± 0.53	85.50 ± 2.38
20200724 (D5)	228.00 ± 14.73	2.03 ± 0.93	11.87 ± 0.25	90.67 ± 0.58
20200727 (D6)	171.67 ± 17.62	1.17 ± 0.45	5.77 ± 0.60	100.00 ± 0.00
20200728 (D7)	123.00 ± 6.98	1.98 ± 0.17	11.95 ± 0.39	86.50 ± 1.29
20200729 (D8)	94.50 ± 5.51	2.58 ± 0.24	11.85 ± 0.26	79.25 ± 1.71
20200730 (D9)	87.25 ± 3.86	2.78 ± 0.26	12.60 ± 0.29	71.00 ± 2.16
20200731 (D10)	60.25 ± 4.35	4.33 ± 0.63	13.50 ± 0.18	71.50 ± 1.29
20200803 (D11)	138.33 ± 5.69	2.40 ± 0.26	10.53 ± 0.38	94.00 ± 2.00

**Table 3:** The results of GLMM on log transformed UFP concentration.

Route	Estimate	Lower	Upper	*t*-Stat	*p*-Value
L1	4.290	4.078	4.502	41.18	$2.49\times 10^{-14}$
L2	4.288	4.076	4.500	41.16	$2.50\times 10^{-14}$
M	3.967	3.755	4.179	38.08	$6.34\times 10^{-14}$

**Table 4:** The parameters of the GLM model on the duration of elevated exposure.

Routes	Estimate	Lower	Upper	z-Value	*p*-Value
L1	2.716	1.802	3.631	5.836	$5.36\times 10^{-9}$
L2	1.858	0.936	2.780	3.959	$7.54\times 10^{-5}$
M	−1.246	−2.417	−0.075	−2.089	0.0367

## Data Availability

Data are available through a supplementary file.

## Supplementary Materials

The following are available online at https://www.mdpi.com/article/10.3390/ijerph18157808/s1, Data.
